# Increased Risk of Second Squamous Cell Carcinomas Following Cervical Cancer: A Nationwide Danish Case–Control Study

**DOI:** 10.1002/cam4.71559

**Published:** 2026-01-27

**Authors:** Sara Bønløkke, Jan Blaakær, Torben Steiniche, Maria Iachina

**Affiliations:** ^1^ Department of Clinical Medicine Aarhus University Aarhus N Denmark; ^2^ Department of Obstetrics and Gynecology Gødstrup Regional Hospital Herning Denmark; ^3^ Department of Obstetrics and Gynecology Odense University Hospital Odense C Denmark; ^4^ Department of Clinical Research University of Southern Denmark Odense M Denmark; ^5^ Department of Clinical Epidemiology Odense University Hospital Odense C Denmark

**Keywords:** cervical cancer survivors, human papilloma virus, second primary cancers, squamous cell carcinoma

## Abstract

**Introduction:**

This nationwide case–control study investigated the risk of second primary cancers among cervical cancer (CC) survivors compared to cancer‐free women.

**Methods:**

Women aged ≥ 18 diagnosed with CC from 1987 to 2012 were identified via the Danish Cancer Registry (DCR) and matched 1:5 by age and residence to cancer‐free controls. A subpopulation with histologically confirmed squamous cell carcinoma of the cervix (SCC‐C) was defined using the Danish Pathology Register. Adjusted sub‐hazard ratios (aSHR) for subsequent primary cancers were estimated.

**Results:**

The study included 10,728 CC cases and 53,597 matched controls, including 7910 SCC‐C cases and 39,358 controls. Over a median follow‐up of 14.2 years, CC survivors had a modest but statistically significant reduction in the overall risk of second primary cancers (aSHR: 0.87; 95% CI, 0.81–0.93), including lower risks of colorectal cancer (aSHR: 0.77; 95% CI, 0.63–0.93), breast cancer (aSHR: 0.76; 95% CI, 0.67–0.87), and malignant melanoma (aSHR: 0.59; 95% CI, 0.42–0.84). In contrast, the risk of lung cancer was increased (aSHR: 1.33; 95% CI, 1.15–1.54). Among SCC‐C survivors, the risk of second SCC was increased at both HPV‐related sites (aSHR: 1.78; 95% CI, 1.18–2.68) and non‐HPV sites (aSHR: 2.59; 95% CI, 1.92–3.47), primarily due to a marked increased risk of lung SCC (aSHR: 2.88; 95% CI, 1.97–4.20).

**Discussion:**

Although the overall risk of second primary cancers was lower among CC survivors compared to controls, the increased incidence of second SCCs, particularly lung SCC, highlights the need for targeted long‐term surveillance strategies.

**Abbreviations**
AC: adenocarcinoma
aSHR: adjusted sub‐hazard ratio
CC: cervical cancer
CCI: charlson comorbidity index
CCS: cervical cancer survivors
CNS: brain/central nervous system
CPR: civil registration numbers
DCR: danish cancer registry
DCRS: danish civil registration system
DNPR: danish national patient registry
DPR: danish pathology register
HPV: human papillomavirus
HR: high‐risk
ICD: international classification of disease
LDCT: low‐dose computed tomography
M: morphological axis
SCC: squamous cell carcinoma
SCC‐C: squamous cell carcinoma of the cervix
SHR: sub‐hazard ratio
SIR: standardized incidence ratio
SNOMED: systematized nomenclature of medicine
T: topographic axis

## 1 Introduction

Persistent infection with high‐risk (HR) human papillomavirus (HPV) is the primary cause of most cervical cancer (CC) cases [1]. Squamous cell carcinoma (SCC) accounts for approximately 82% of CCs, adenocarcinoma (AC) about 12%, and other less common subtypes account for the remaining [2]. The strong association between SCC and HPV has been well established since 1977, where high‐risk HPV (HR‐ HPV) has been detected in 99.7% of cases with squamous cell carcinomas of the cervix (SCC‐C) [3]. Nevertheless, HPV infection does not account for approximately 15% of AC cases [4].

In Western countries, the 5‐year overall survival rate for CC across all stages ranges between 70% and 90% [5, 6]. Given this survival rate, it is important to assess the risk of second primary cancers among patients previously treated for CC. Epidemiological studies show that CC survivors have a higher incidence of subsequent primary cancers compared to the general population [7]. Although many studies have examined second cancers following radiotherapy for CC [8, 9], more research is needed on HPV‐related cancers, both at anatomical sites commonly associated with HPV but not least at sites not traditionally linked to HPV.

HPV infections in the cervix often coincide with infections in the oropharyngeal and anogenital regions, likely because of shared transmission routes [10, 11]. Persistent HR‐HPV infection in these areas can lead to premalignant lesions like those in the cervix, increasing the risk of malignant transformation [12, 13]. As a recognized oncogenic driver, HPV is closely associated with the development of SCCs of the vulva, vagina, anus, tonsils, and base of the tongue [7, 14]. Therefore, it is not unexpected that previous studies have found an increased risk of cancers at these HPV‐related sites in women treated for CC [15]. Furthermore, emerging evidence suggests that HPV may also contribute to SCC carcinogenesis in other anatomical regions, including the esophagus, oral cavity, larynx, hypopharynx, and lungs, but its etiological role remains controversial [16].

Denmark's tax‐funded healthcare system offers a distinctive setting for exploring the risk of second primary cancers among CC survivors. Using comprehensive nationwide health registries, enabled by unique civil registration numbers (CPR) [17, 18], allows for high‐quality, population‐based epidemiological research.

This case–control study, based on histologically confirmed diagnoses, aimed to assess whether a previous diagnosis of CC increases the risk of developing new primary cancers over time. Particular attention is given to SCCs, including those occurring at anatomical sites not traditionally linked to HPV. Furthermore, this study examines the hypothesis that HPV infection may play a role in one or more of the five most prevalent cancers in Denmark, namely breast, lung, colorectal, malignant melanoma, and brain/central nervous system (CNS) cancers.

## 2 Methods

This nationwide population‐based cohort study includes a subset of the study population described previously [19]. It comprises a case and a control group.

### 2.1 Total Study Population

Cases included all women aged 18 years or older diagnosed with CC between 1st of January 1987 and 31st of December 2012, identified through the Danish Cancer Registry (DCR) [20]. Controls were selected from the Danish Civil Registration System (DCRS) at a 5:1 ratio, using traditional individual matching by birth year, date (±7 days), and residence at the index date. To be eligible, controls had to be alive and living in Denmark at the time of the CC diagnosis of the matched case, have no prior CC diagnosis in the DCR, and be matched to only one case.

Non‐cervical cancers diagnosed following the index date were identified through the DCR, with follow‐up data available up to 6th of October 2018, ensuring a minimum follow‐up period of 5 years and 9 months. Consequently, all women were followed from the index date until the first occurrence of a new cancer diagnosis, death, emigration, or October 2018, whichever came first. Non‐melanoma skin cancers (Table S1) were excluded from the analysis because of incomplete registration in cancer and healthcare registries across most countries [21, 22, 23].

Comorbidity data up to ten years pre‐index date were obtained from the Danish National Patient Registry (DNPR) and classified using the Charlson Comorbidity Index (CCI) (categorized as low [0], moderate [1 or 2], or high [> 2]) [24]. For consistent cancer diagnoses, we cross‐referenced CC cases from the DNPR with the DCR, only including cases listed in both. DNPR has recorded inpatient consultations since 1977 and outpatient consultations since 1995 [25].

**FIGURE 1:**
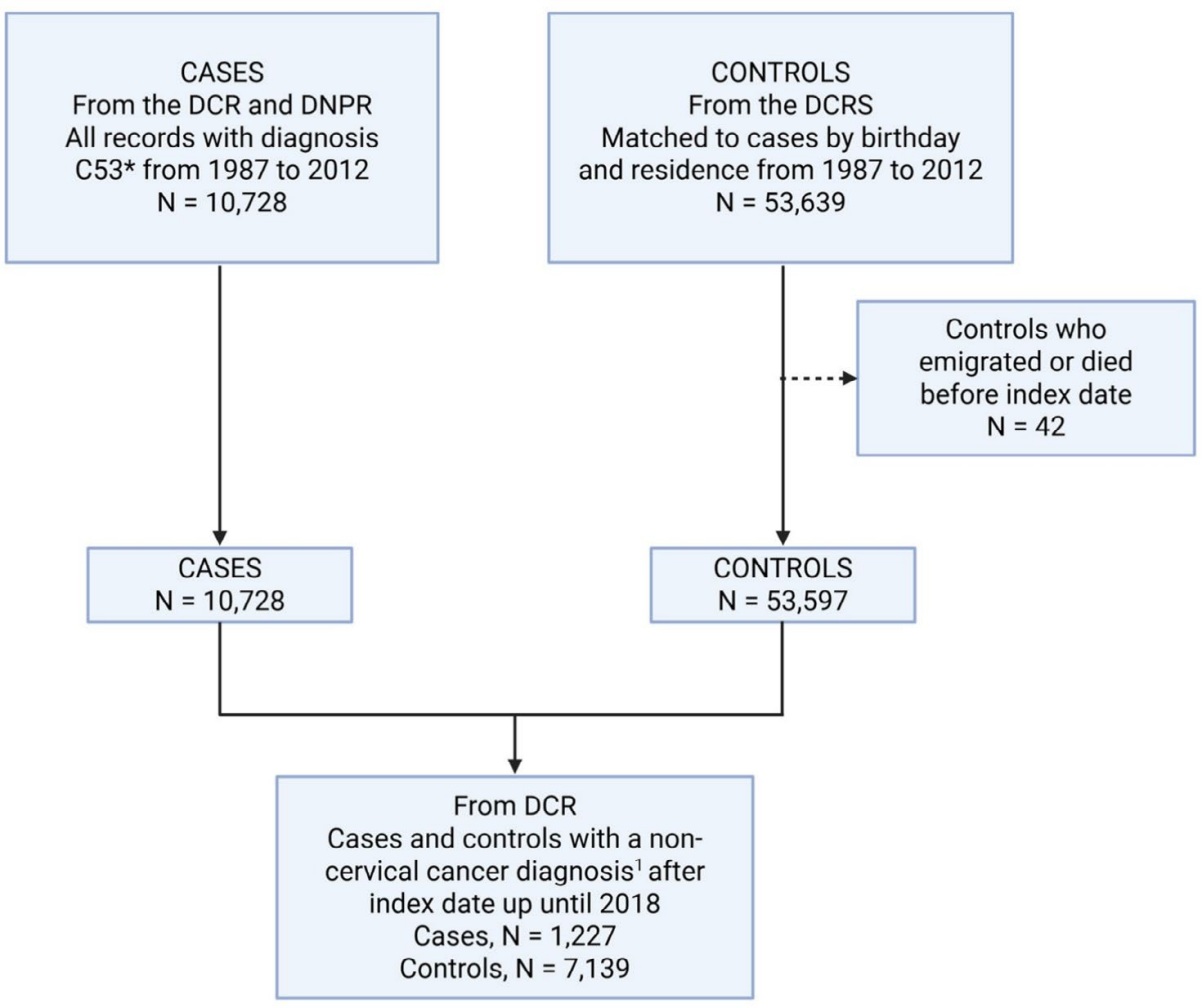
Total study population. Except for non‐melanoma skin cancer (NMSC)^1^.

The final study population included all cases with histologically confirmed CC and their matched controls (Figure 1).

### 2.2 Squamous Cell Subpopulation

To explore a potential association between HPV and non‐cervical SCC, we used the Danish Pathology Register (DPR) to identify all histologically confirmed SCCs diagnosed in cases and controls after the index date, up to 6th of October 2018. Since 1977, Danish pathology departments have recorded patho‐anatomical data [26] using a national adaptation of the Systematized Nomenclature of Medicine (SNOMED) [27]. Morphology (M) codes were used to confirm that tumors were primary rather than metastatic and to ensure that only SCCs were included (Table S2). For women with multiple records within 180 days, topography (T) codes were applied to determine the anatomical site of the tumor. Furthermore, to ensure alignment between cancer diagnoses in the DCR and the DPR, we excluded cases without a corresponding SCC diagnosis in the DPR within ±180 days of the DCR‐recorded diagnosis along with their corresponding controls.

**FIGURE 2:**
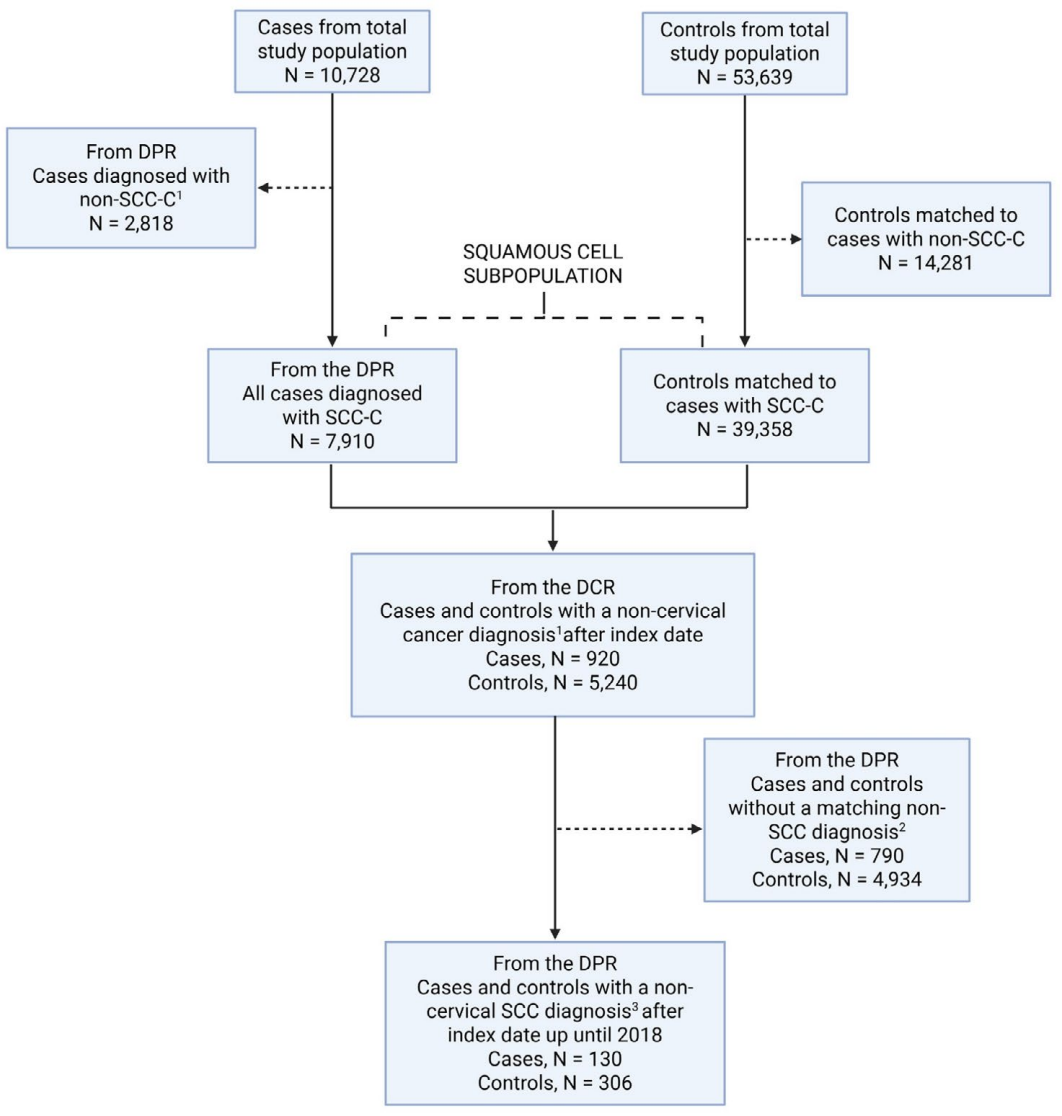
Squamous cell subpopulation. ^1^Except for NMSC. ^2^No record of a cancer diagnosis with a morphological SNOMED code for SCC (M805D3, M80703, M80713, M80723, M80733, M80743, M80753, M80763, M8076X, M80783, M807F3, M807K3, M80833, M80843, M80853, M80863, M80943, M80953 (see Table S1)) ±180 days of the cancer diagnosis in DCR. ^3^Cancer diagnosis with morphological SNOMED codes outlined in ^2^ and Table S2.

The final squamous cell subpopulation included cases with histologically confirmed SCC‐C and their corresponding matched controls (Figure 2).

Like for the total study population, non‐cervical SCC diagnosed following the index date were identified through DCR, with follow‐up data available up to 6th of October 2018. We divided non‐cervical SCCs into two subgroups: HPV related SCCs comprising cancers of the vulva, vagina, anus, and oropharynx, which have a known or potential link to HPV (Table S4), and non‐HPV related SCCs, which have no established association with HPV.

### 2.3 Statistical Analyses

STATA version 18 (StataCorp, College Station, TX) was used for all statistical analyses. To assess the association between group (cases vs. controls) and the risk of other primary cancer, we used the competing risks approach. This approach treated both death and the occurrence of other primary cancers as competing risks. We estimated unadjusted and adjusted sub‐hazard ratio (SHR) with 95% Confidence Intervals (CI). The adjusted models accounted for potential confounders, including CCI at index date, year of birth, residence, disease stage at diagnosis (i.e., localized disease vs. regional disease or distant metastases according to ICD‐10 [28]), age, education, working status, civil status, and socio‐economic status.

Analyses were conducted for all other cancer diagnoses, all other SCCs, and stratified by cancer localization, i.e., HPV related and non‐HPV related SCCs, respectively. A *p*‐value < 0.05 was considered statistically significant.

## 3 Results

### 3.1 Selection of the Total Population and the Squamous Cell Subpopulation

The total study population included 10,728 CC cases and 53,639 controls (Figure 1). Based on data from DPR, 2818 cases were classified as non–squamous cell carcinoma (non–SCC‐C, primarily ACs). This resulted in a final squamous cell subpopulation comprising 7910 cases and 39,358 matched controls (Figure 2).

### 3.2 Risk of Non‐Cervical Cancer in Cases and Controls

**FIGURE 3:**
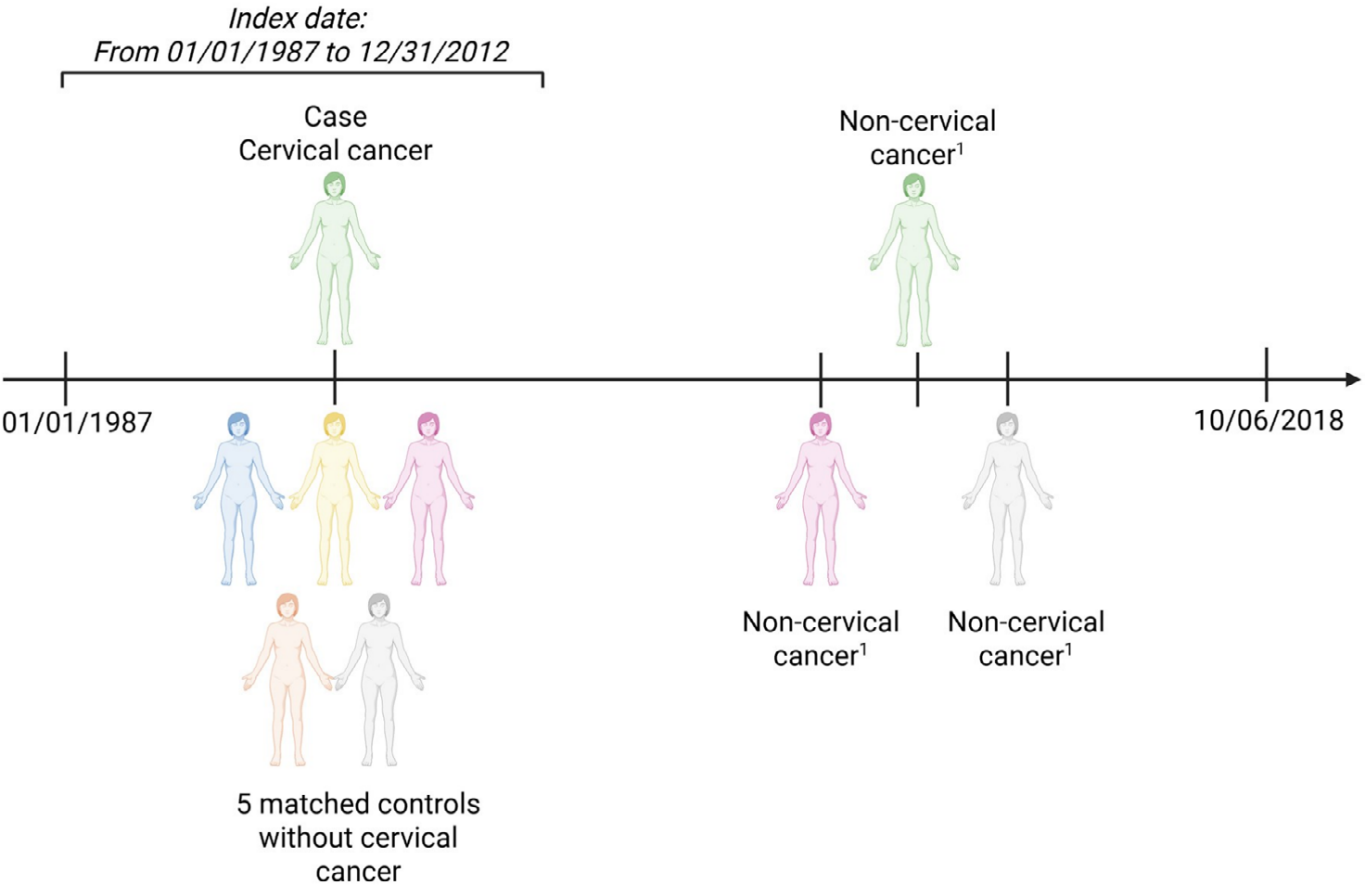
Illustration of the data collection in cases and matched controls. Non‐cervical cancers are all other cancer diagnoses than CC in the total study population and all other squamous cell cancers in squamous cell subpopulation^1^.

In the total study population, we evaluated the prevalence of all non‐cervical cancers diagnosed after the index date among cases and controls, with follow‐up until October 2018. This corresponded to a median follow‐up duration of 13.5 years and a minimum of 5 years and 9 months. Likewise, in the squamous cell subpopulation, we assessed the prevalence of non‐cervical SCCs under the same follow‐up conditions, and here, median follow‐up was 14.2 years. Figure 3 illustrates the identification process for these cancer records and the associated follow‐up period.

### 3.3 Analyses From the Total Study Population

In the total study population, 1227 cases and 7139 controls were registered in the DCR with a diagnosis of non‐cervical cancer after the index date (Figure 1). For the analysis of overall non‐cervical cancer risk, only the first subsequent cancer diagnosis was considered for women with multiple cancer incidences. Across all cancer types, the unadjusted SHR for cases versus controls was 0.76 (95% CI, [0.71–0.81]), and the adjusted SHR (aSHR) was 0.87 (95% CI, [0.81–0.93]) (Table 1), showing a modest but statistically significant reduction in the risk of non‐cervical cancers among women with CC, regardless of histological type.

**TABLE 1:** Total study population: SHR (sub‐hazard ratio) and 95% confidence interval (95% CI) for non‐cervical cancers estimated by competing risk analyses.

Cases with all histological types of CC vs matched controls^a^	Cases *N* = 10,728 *N* (%)	Controls *N* = 53,367 *N* (%)	Unadjusted SHR (95% CI)	Adjusted SHR (95% CI)^a^
All non‐cervical cancers^b^	1227 (11.5%)	7139 (13.4%)	0.76 (0.71; 0.81)	0.87 (0.81; 0.93)
Specific non‐cervical cancers^c^				
Breast cancer	289 (2.7%)	2022 (3.8%)	0.65 (0.57; 0.73)	0.76 (0.67; 0.87)
Lung cancer	282 (2.6%)	1069 (2.0%)	1.22 (1.07; 1.40)	1.33 (1.15; 1.54)
Colon or rectum cancer	167 (1.6%)	1115 (2.1%)	0.67 (0.57; 0.80)	0.77 (0.63; 0.93)
Malignant melanoma	45 (0.4%)	375 (0.7%)	0.55 (0.40; 0.75)	0.59 (0.42; 0.84)
Cancer of the brain and CNS	18 (0.2%)	89 (0.2%)	0.90 (0.53; 1.54)	0.99 (0.58; 1.73)
Other	484 (4.5%)	2821 (5.3%)	0.74 (0.67; 0.82)	0.85 (0.76; 0.95)

^a^ Adjusted for CCI, stage at diagnosis, age, cohort, education, comorbidity, working status, socio‐economic status, civil status, and country of birth.

^b^ The risk of all non‐cervical cancers except for non‐melanoma skin cancer.

^c^ The five most prevalent female cancer types in Denmark—breast, lung, colorectal, malignant melanoma, and brain/central nervous system cancers—were analyzed alongside a combined group of all other cancer types, excluding non‐melanoma skin cancer. It is important to note that the total number of all non‐cervical cancers is lower than the sum of the individual cancer types reported. This discrepancy arises because some women were diagnosed with more than one cancer after the index date; however, only the first cancer diagnosis was counted in the overall estimate of non‐cervical cancers.

We then evaluated the risk of the five most common female cancer types in Denmark, namely breast, lung, colorectal, malignant melanoma, and CNS cancers [29], as well as a combined group of all other cancer types (ICD‐10 codes listed in Table S3). Compared to controls, cases had a modest but statistically significant decreased risk of breast cancer (aSHR: 0.76 [0.67; 0.87]), colorectal cancer (aSHR: 0.77 [0.63; 0.93]), malignant melanoma (aSHR: 0.59 [0.42; 0.84]), and the grouped category of all other cancer types (aSHR: 0.85 [0.76; 0.95]). Conversely, the risk of lung cancer was significantly increased in cases (aSHR: 1.33 [1.15; 1.54]). No statistically significant differences were observed for CNS cancers (0.99 [0.58; 1.73]) (Table 1).

### 3.4 Analyses From the Squamous Cell Subpopulation

Following this, a subset of the study population, comprising 7910 SCC‐C cases and their 39,358 matched controls, was selected for analysis. By means of the DCR, we identified 920 cases and 5240 controls with a non‐cervical cancer diagnosis after the index date (Figure 2). We focused on non‐cervical cancer diagnoses of squamous cell histology (Table S2), and all other morphological codes were excluded, resulting in 130 cases and 306 controls with a confirmed diagnosis of non‐cervical SCC after the index date (Figure 2).

Again, competing risk analysis was used to compare the risk of non‐cervical SCC between cases with SCC‐C and matched controls before and after adjusting for the same parameters as described previously. As previously, only the first subsequent SCC diagnosis was considered for women with multiple cancer incidences. For all other SCCs, we found an unadjusted SHR of 2.15 (95% CI, [1.75; 2.64]) and an aSHR of 2.26 [1.78; 2.88] between cases and controls, respectively (Table 2). This clearly underlines an increased risk of non‐cervical SCCs in cases compared to controls.

**TABLE 2:** Squamous cell subpopulation: SHR (sub‐hazard ratio) and 95% Confidence Interval (95% CI) for non‐cervical SCCs with and without a known association with HPV estimated by competing risk analysis.

	Cases *N* = 7910 *N* (%)	Controls *N* = 39,403 *N* (%)	Unadjusted SHR (95% CI)	Adjusted SHR (95% CI)^a^
Non‐cervical SCCs				
All non‐cervical SCCs	130 (1.6%)	306 (0.8%)	2.15 (1.75; 2.64)	2.26 (1.78; 2.88)
HPV related SCCs^b^				
Total	40 (0.5%)	123 (0.3%)	1.64 (1.15; 2.34)	1.78 (1.18; 2.68)
Vulvar or vaginal cancer	17 (0.2%)	40 (0.1%)	2.14 (1.22; 3.78)	2.40 (1.19; 4.84)
Anal cancer	10 (0.1%)	34 (0.1%)	1.48 (0.73; 3.00)	1.54 (0.71; 3.31)
Oropharyngeal cancer	14 (0.2%)	45 (0.1%)	1.57 (0.86; 2.86)	1.83 (0.94; 3.59)
Non‐HPV related SCCs^c^				
Total	90 (1.1%)	183 (0.5%)	2.49 (1.93; 3.20)	2.59 (1.92; 3.47)
SCC of the lung	58 (0.7%)	106 (0.3%)	2.76 (2.00; 3.80)	2.88 (1.97; 4.20)

^a^ Adjusted for CCI, stage at diagnosis, age, cohort, education, comorbidity, working status, socio‐economic status, civil status, and country of birth. Be aware that the total number of HPV related SCCs is lower than the combined total of vulvar/vaginal, anal, and oropharyngeal cancers. This is because some women developed more than one HPV related SCC after the index date, but these cases only counted for one in the total estimate.

^b^ HPV related SCCs are SCCs with a known association with HPV, see Table S3.

^c^ Non‐HPV related SCCs are SCCs with no known association with HPV.

After categorization of non‐cervical SCCs into HPV related SCCs (Table S4) and non‐HPV related SCCs, we conducted competing risk analyses to compare the risk of HPV related and non‐HPV related SCCs between cases and controls.

#### 3.4.1 Risk of HPV Related Non‐Cervical SCCs in the Squamous Cell Subpopulation

Overall, a significantly increased risk of HPV related SCC was observed among cases, with an aSHR of 1.78 [95% CI, 1.18–2.68]. Because of limitations in distinguishing between vulvar and vaginal cancer topographies in the DPR, these sites were combined into a single category. For vulvar or vaginal cancer, aSHR was 2.40 [1.19–4.84], while aSHRs for anal cancer and oropharyngeal cancer were 1.54 [0.71–3.34] and 1.83 [0.93–3.59], respectively, suggesting increased risks that did not reach statistical significance.

#### 3.4.2 Risk of Non‐HPV Related Non‐Cervical SCCs in the Squamous Cell Subpopulation

**FIGURE 4:**
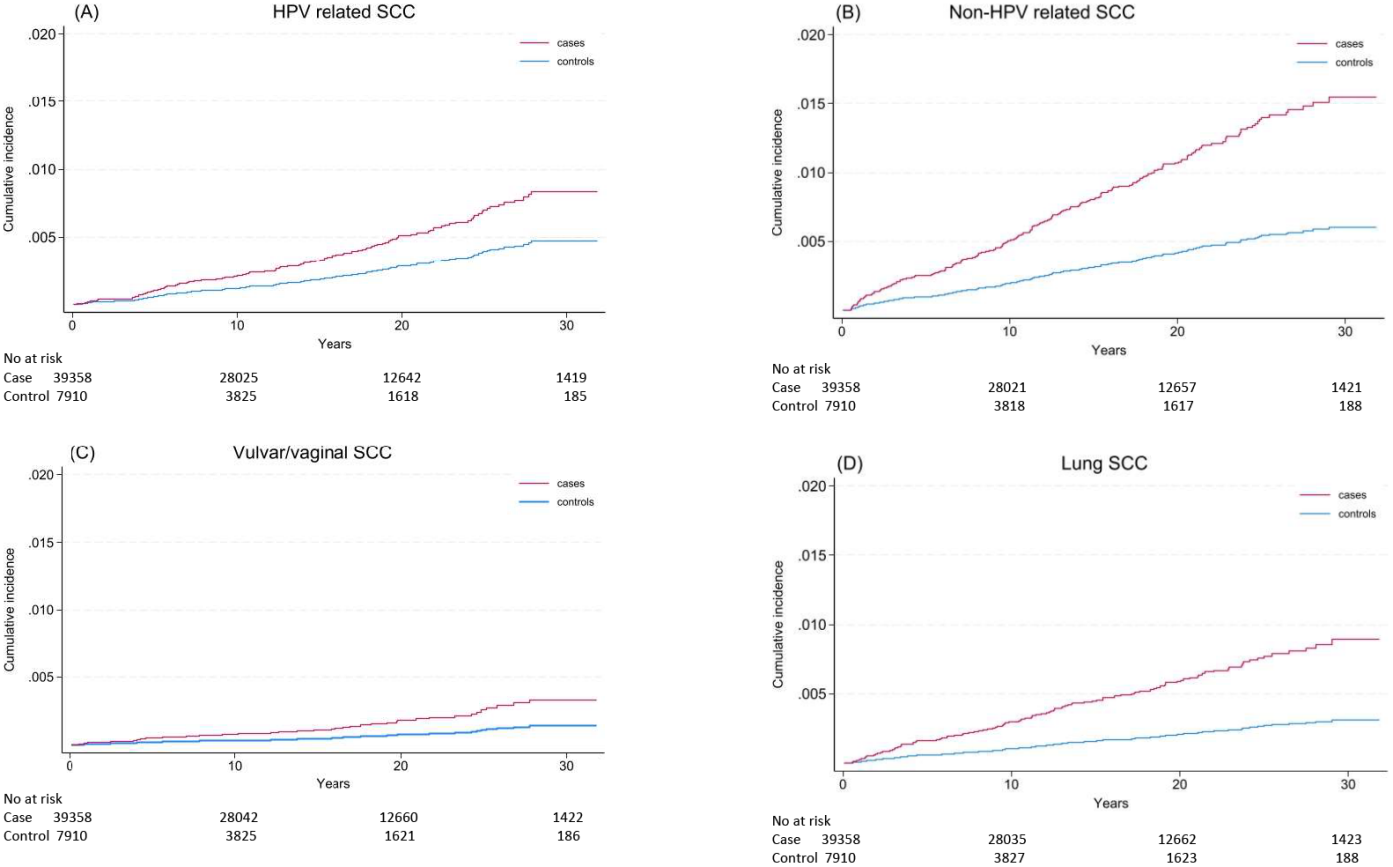
Thirty‐year cumulative incidence of HPV‐related squamous cell carcinomas (A), non‐HPV‐related squamous cell carcinomas (B), as well as SCCs of the vulva/vagina (C) and lung (D).

We found a significantly increased risk of SCCs at sites not typically linked to HPV (i.e., non‐HPV related SCC), with an aSHR of 2.59 [1.92; 3.47] (Table 2 and Figure 4). This was largely driven by SCC of the lungs (aSHR 2.88 [1.97; 4.20]) (Table 2).

## 4 Discussion

In this nationwide case–control study, we used Danish registry data to assess the risk of second primary cancers in women with a history of CC. Over a median follow‐up of 13.5 years, we observed a modest but statistically significant overall reduction in cancer risk with an estimated aSHR of 0.87. Among the five most common cancers in Danish women, CC survivors had an increased risk of lung cancer, while the risks of colorectal cancer, breast cancer, and malignant melanoma were significantly reduced.

Among women with a history of SCC‐C, the risk of subsequent SCCs was significantly increased (aSHR: 2.26), with increased risks observed at both HPV‐related sites (aSHR: 1.78) and non‐HPV‐related sites (aSHR: 2.59). The increased risk at non‐HPV‐related sites was primarily driven by SCC of the lung (aSHR: 2.88).

The modest but statistically significant decrease in the risk of second primary cancers over time represents a novel finding that contrasts with most previous population‐based studies, showing an overall increased risk of second primary cancers among women with prior CC [7, 30, 31]. In contrast to our matched case–control design with adjusted HRs, these studies rely on standardized incidence ratios (SIRs), comparing observed cancers in CC survivors with the expected number in the general female population. Consequently, estimates may be influenced by differences in competing mortality, demographic composition, and underlying incidence rates, thereby preventing a direct comparison of absolute risks between women with and women without prior CC. Contrary, our matched case–control design enabled a direct estimation of second primary cancer incidence relative to a demographically similar control group. Thus, our finding of an overall slightly reduced risk is therefore not necessarily inconsistent with the previous literature based on SIRs but may reflect methodological differences.

A notable finding in our study is the significantly increased risk of lung cancer over time among CC survivors. The aSHR for lung cancer across all histological types was 1.33 (95% CI, 1.15–1.54), increasing markedly to 2.88 (95% CI, 1.97–4.20) when restricted to SCC of the lung. Supporting this observation, a comprehensive review by Parama et al. [32] highlights growing evidence for a potential role of HPV infection in lung carcinogenesis. More recent studies further strengthen the possibility of a causal association, particularly for certain histological subtypes such as SCC. A mendelian randomization study reported that HPV16 infection was significantly associated with increased risk of lung SCC (OR = 7.69; 95% CI, 1.98–29.85) [33]. Also, findings from two meta‐analyses indicate strong associations of HPV16/18 with lung SCC (OR = 9.78; 95% CI, 6.28–15.22) [34] and an overall increased risk of lung cancer in HPV positive individuals (pooled OR = 3.64; 95% CI, 2.60–5.08), with a stronger association observed among non‐smokers [35]. This suggests that HPV may play a causal role in lung cancer development, especially in individuals without strong risk factors like smoking, indicating that the increased lung cancer risk among CC survivors is unlikely to be explained solely by shared risk factors, such as smoking.

Our study design, which limited inclusion to specific morphological codes for primary SCC diagnoses (Table S2), naturally excluded lung cancers diagnosed solely based on metastatic samples. Although this may cause an underestimation of the true incidence of lung cancer, the impact is likely non‐differential between cases and controls.

We found a decreased risk of breast cancer over time among women with a history of CC with an aSHR of 0.76. However, existing literature on this association remains somewhat inconsistent. While some studies indicate an increased risk [16], others report a reduced risk [36, 37, 38]. In Denmark, more than half of CC patients receive combined chemoradiation, a treatment that may reduce breast cancer risk by inducing ovarian failure and thereby lowering estrogen levels. This finding aligns with previous studies [38, 39, 40]. Boice et al. [39] reported a 44% reduction in breast cancer risk following high‐dose ovarian radiation, and the Danish cohort study by Koch et al. [40] showed that bilateral oophorectomy was associated with a significantly lower incidence of breast cancer (adjusted rate ratio 0.79; 95% CI, 0.64–0.99).

The present study showed a statistically significant reduction in the risk of malignant melanoma over time among women with a history of CC (aSHR: 0.59). This finding is consistent with a large population‐based study involving approximately 95,000 CC cases from cancer registries in Denmark, Finland, Norway, Sweden, and the United States [36], which reported a modest but significant decrease in melanoma risk among SCC‐C survivors. Increased health awareness may explain the observed reduction, potentially promoting protective behaviors such as reduced UV exposure and more frequent sunscreen use. Studies have shown that a cancer diagnosis is often associated with shifting toward healthier lifestyle practices [41]. Similarly, a large cohort study on second primary malignant melanomas by Yang et al. [42] found a reduced melanoma risk among cancer survivors and suggest that this may reflect general survivorship behaviors and risk modifications. Besides, it is likely that socioeconomic differences and increased health awareness contribute to this decreased risk.

Surprisingly, we observed a small but statistically significant reduction in colorectal cancer among our cohort of CC survivors. Most large population‐based studies report an increased risk of second primary colorectal cancer, particularly after pelvic radiotherapy [7, 43, 44, 45], with these cancers typically occurring 8–15 years post‐treatment [43, 44, 45, 46]. However, similar to our findings, one study by Lim et al. [38] found a lower incidence of rectal and rectosigmoid junction cancers among CC survivors, although this reduction reached significance only within the first 60 months of follow‐up in women aged ≥ 50 years. Taken together with our findings, this highlights the need for long‐term follow‐up (> 15 years) to fully capture the true risk of radiation‐induced colorectal cancer.

We observed an overall increased risk over time of second primary SCCs at HPV‐related sites, including the vagina/vulva, anus, and oropharynx, among women with prior SCC‐C with an aSHR of 1.78. When analyzing these sites individually, only vulvar/vaginal cancer showed a statistically significant association with an aSHR of 2.40. No significant associations were found for anal cancer or oropharyngeal cancer, which may reflect limited statistical power because of the rarity of these cancers. In agreement with this, previous studies show robust evidence for an overall increased risk over time of second primary SCCs at HPV‐related sites, including the vagina, vulva, anus, and oropharynx, among women with prior CC [36, 47, 48, 49, 50].

As discussed in the introduction, cervical HPV infections often co‐occur with infections at other mucosal sites, such as the oropharynx and anogenital regions, likely because of shared transmission pathways, including sexual contact [10, 11]. Women with a history of CC may possess host‐related risk factors, such as genetic polymorphisms [51], dysregulation of immune and innate defense mechanisms [52], and alterations in the microbiome [53], that increase susceptibility to HPV infection and persistence. These biological predispositions may increase the risk of developing additional HPV‐related malignancies.

Given the significantly increased risk of both HPV‐related cancers and cancers not previously associated with HPV (non‐HPV related) among women with a history of SCC‐C, there is a critical need for improved prevention and early detection strategies in this population. However, the absence of validated screening tools currently limits these efforts. For example, no standardized approaches, such as vault smears or colposcopic inspections, exist for the early detection of vaginal and vulvar cancers in hysterectomized women. Similarly, routine use of anal cytology and anoscopic screening for anal cancer remains uncommon [54, 55].

Considering the significantly increased risk of second primary lung cancer among CC survivors, routine screening using low‐dose computed tomography (LDCT) may provide additional clinical benefits. The United States Preventive Services Task Force currently recommends annual LDCT screening for individuals aged 50 to 80 years with a 20 pack‐year smoking history who currently smoke or have quit within the past 15 years [56]. While smoking risk primarily underpins these guidelines, the increased incidence of lung cancer in CC survivors suggests a need for more tailored screening protocols.

While HPV may contribute to cancers of the lung, esophagus, and head and neck, the increased risk for these cancers in patients treated for CC may also reflect shared risk factors like smoking [57, 58, 59]. Given that smoking rates are higher among CC patients compared to the general population, targeted lifestyle interventions, especially tobacco cessation support, could be beneficial [59].

### 4.1 Strengths and Limitations

This nationwide case–control study benefits from a large sample size, long follow‐up period, and virtually complete tracking using unique CPR numbers. Linking DCR with the DPR improved diagnostic accuracy through histologically confirmed data. The matched case–control design provides better control of confounding compared to population‐based studies. By matching cases and controls on birth year, date (±7 days), and residence at index date, we ensured that CC cases were compared with women of similar background characteristics, thereby reducing bias from these important determinants of cancer risk. This approach may offer a somewhat more comparable reference than population‐based designs using the general female population. Furthermore, it allows estimation of adjusted hazard ratios, whereas population‐based studies generally rely on standardized incidence ratios. Nevertheless, we acknowledge that population‐based cohort studies have complementary strengths, particularly with regard to representativeness and generalizability, and that both designs provide valuable but somewhat different perspectives.

Furthermore, we acknowledge that detection bias remains a potential limitation in our study. While information on follow‐up medical checkups after CC treatment would have been valuable, such data are not available in the registers we used. Differences in surveillance intensity between cases and controls may therefore have influenced the observed incidence of second primary cancers. It is also plausible that treatment modality, that is surgery versus chemoradiation, affects the subsequent risk of developing second primary cancers. In Denmark, treatment is strongly stage‐dependent, with localized disease generally managed surgically and advanced disease treated with chemoradiation [60]. As our adjusted analyses included “stage at diagnosis,” we believe that we have, at least partly, taken this into account, although we cannot rule out some residual confounding related to treatment. Furthermore, the observed decreased risk over time of several cancers shows that the influence of such bias may be limited.

A key limitation of this study is the lack of data on HPV type in CC cases and other anogenital cancers. To enhance specificity for HPV related cancers, we restricted the analysis to SCCs. Nonetheless, it is important to note that HPV causally links to only 20%–50% of vulvar cancers, 60%–65% of vaginal cancers, 83%–95% of anal cancers, and 51%–71% of oropharyngeal cancers [61]. Consequently, our results do not provide direct evidence that HPV was the causal factor in the subsequent development of vulvar, vaginal, anal, or oropharyngeal cancers in either cases or controls.

## 5 Conclusion

In this nationwide, population‐based case–control study using Danish registry data, we evaluated the long‐term risk of second primary cancers among women with a history of CC. We furthermore hypothesized that HPV infection may contribute to the development of the five most prevalent cancers in Denmark. Over a median follow‐up of 13.5 years, we observed a modest but statistically significant overall reduction in the risk of non‐cervical cancers. Notably, the risks of breast cancer, colorectal cancer, and malignant melanoma were reduced, while the risk of lung cancer increased. Given the high background incidence of breast cancer and melanoma in Denmark, these site‐specific reductions may have contributed significantly to the overall lower second cancer risk among CC survivors.

In contrast, among women with prior SCC‐C, we observed a marked increase in the risk of developing subsequent SCCs, both at HPV‐related and HPV‐unrelated sites. The strongest contributor was SCC of the lung, highlighting a potential causal role of HPV in the development of lung SCC.

These findings underscore the need for targeted long‐term surveillance strategies for CC survivors, including attention to second primary cancers not traditionally linked to HPV.

## Author Contributions

**Sara Bønløkke, Jan Blaakær, Torben Steiniche**, and **Maria Iachina:** conceptualization, methodology, validation, and writing – review and editing. **Sara Bønløkke**, and **Maria Iachina:** data curation and formal analysis. **Sara Bønløkke:** funding acquisition, project administration, and resources. **Sara Bønløkke, Torben Steiniche**, and **Maria Iachina:** investigation and visualization. **Maria Iachina:** software development. **Jan Blaakær, Torben Steiniche**, and **Maria Iachina:** supervision. **Sara Bønløkke**, and **Torben Steiniche:** writing – original draft.

## Funding

This study was funded by IMK Almene Fond (30–206‐343).

## Consent

This study was approved by the Danish Patient Safety Authority (record number 31–1521‐438) and is registered in the internal register of research projects at Central Denmark Region (record number 1–16–02‐316‐20) and at Aarhus University (record number 2016–051‐000001). According to Danish legislation, registry‐based research does not require approval from the Ethics Committee, nor does it require informed consent from participants.

## Conflicts of Interest

The authors declare no conflicts of interest.

## Supporting information


**Table S1:** ICD‐10^1^ codes of non‐melanoma skin cancer (NMSC) excluded due to incomplete registration.
**Table S2:** Morphological (M) SNOMED codes1 for SCCs.
**Table S3:** Diagnostic codes for the five most prevalent female cancer types in Denmark according to International Classification of Diseases 10th revision (ICD‐10)1 for SCCs.
**Table S4:** ICD‐101 codes from DCR and SNOMED2 codes from DPR for non‐cervical SCCs with a known association with HPV.

## Data Availability

The data used in this study are available from Statistics Denmark under license and are not publicly accessible. Access may be granted upon reasonable request and with approval from the Danish Health Data Authority (contact: Anna Ruback Birkmose, anba@sundhedsdata.dk) and Statistics Denmark (contact: Marianne Andresen, mia@dst.dk).
