# Supplementary material for: Increased Risk of Second Squamous Cell Carcinomas Following Cervical Cancer: A Nationwide Danish Case–Control Study
Source: Cancer Med. 2026 Jan 27;15(2):e71559. doi: 10.1002/cam4.71559 (PMC12835779; doi:10.1002/cam4.71559)
Supplement: Supplementary file 1 — Table S1: ICD‐101 codes of non‐melanoma skin cancer (NMSC) excluded due to incomplete registration. Table S2: Morphological (M) SNOMED codes1 for SCCs. Table S3: Diagnostic codes for the five most prevalent female cancer types in Denmark according to International Classification of Diseases 10th revision (ICD‐10)1 for SCCs. Table S4: ICD‐101 codes from DCR and SNOMED2 codes from DPR for non‐cervical SCCs with a known association with HPV. [file CAM4-15-e71559-s001.docx]

Supplementary material

**Increased Risk of Second Squamous Cell Carcinomas Following Cervical Cancer: A Nationwide Danish Case-Control Study**

Sara Bønløkke^a, b^, Jan Blaakær^c, d^, Torben Steiniche^a^, Maria Iachina^e^

^a^ Department of Clinical Medicine, Aarhus University, Aarhus N, Denmark,

sarasim@clin.au.dk, steiniche@clin.au.dk

^b^ Department of Obstetrics and Gynecology, Gødstrup Regional Hospital, Herning, Denmark

^c^ Department of Obstetrics and Gynecology, Odense University Hospital, Odense C, Denmark, jan.blaakær@rsyd.dk

^d^ Department of Clinical Research, University of Southern Denmark, Odense M, Denmark

^e^ Department of Clinical Epidemiology, Odense University Hospital, Odense C, Denmark, maria.iachina@rsyd.dk

**Table S1. ICD-10^1^ codes of non-melanoma skin cancer (NMSC) excluded due to incomplete registration:**

| ICD-10 code | Denotation |
| --- | --- |
| DC440 | Other skin cancer on the lip |
| DC441 | Other skin cancer on eye lid |
| DC442 | Other skin cancer on the ear of the external ear canal |
| DC443 | Other skin cancer in the face with other or non-specified location |
| DC444 | Other skin cancer on scalp or neck |
| DC446 | Other skin cancer on the upper extremity |
| DC447 | Other skin cancer on the lower extremity |
| DC448 | Other skin cancer affected multiple locations |
| DC449 | Other skin cancer |

Please notice that DC445 is not included in this Table, since this code denotes “Other skin cancer on the body” with DC445A denoting “Other skin cancer in anus”, DC445B denoting “other skin cancer of the breast”, and DC445C denoting “Other skin cancer in perineum”. Anal cancer is known to be associated with HPV. Nevertheless, based on the data provided by DCR, differentiation between DC445A, DC445B, and DC445C was not possible. Therefore, to prevent the exclusion of anal cancer cases from our analysis, the DC445 code was included in our dataset.

^1^ According to Fritz APC et al. International classification of diseases for oncology (ICD-O). 3rd ed. Geneva: WHO; 2000.

**Table S2. Morphological (M) SNOMED codes^1^ for SCCs.**

| M code | Denotation |
| --- | --- |
| M805D3 | Squamous cell carcinoma, desmoplastic type |
| M80703 | Squamous cell carcinoma |
| M80713 | Squamous cell carcinoma, keratinizing |
| M80723 | Squamous cell carcinoma, non-keratinizing |
| M80733 | Squamous cell carcinoma, small-cell |
| M80743 | Squamous cell carcinoma, spindle cell type |
| M80753 | Adenoid squamous cell carcinoma |
| M80763 | Micro invasive squamous cell carcinoma |
| M8076X | Micro invasive squamous cell carcinoma, OBS PRO |
| M80783 | Squamous cell carcinoma, basaloid type |
| M807F3 | Squamous cell carcinoma, poorly differentiated |
| M807K3 | Squamous cell carcinoma on the basis of immune profile |
| M80833 | Squamous cell carcinoma, poorly differentiated |
| M80843 | Squamous cell carcinoma, clear cell type |
| M80853 | Squamous cell carcinoma, HPV positive |
| M80863 | Squamous cell carcinoma, HPV negative |
| M80943 | Mixed adeno- and squamous cell carcinoma |
| M80953 | Meta typical carcinoma |

^1^ SNOMED codes according to *Kodebog for patologisk-anatomiske undersøgelser. 3rd edn. .* National Board of Health, 1996.

**Table S3. Diagnostic codes for the five most prevalent female cancer types in Denmark according to International Classification of Diseases 10^th^ revision (ICD-10)^1^ for SCCs.**

| ICD-10 code^1^ | Denotation |
| --- | --- |
| DC50* | Breast cancer |
| DC34*+DC45* | Lung cancer |
| DC18*+DC20* | Colon or rectum cancer |
| DC70*+DC71*+DC72* | Brain cancer |

^1^ According to Fritz APC et al. International classification of diseases for oncology (ICD-O). 3rd ed. Geneva: WHO; 2000.

**Table S4. ICD-10^1^ codes from DCR and SNOMED^2^ codes from DPR for non-cervical SCCs with a known association with HPV**

| HPV-associated cancers | ICD-10 code^1^ and denotation | SNOMED code^2^ and denotation |
| --- | --- | --- |
| Vulva | DC518 – Cancer of the external female genitals affecting multiple locations  DC519 – Cancer of the external female genitals | T02511 – Skin on vulva  T0251C – Skin on vulva – right side  T0251D – Skin on vulva – left side  T02503 – Skin on pubis  T02520 – Skin on labium  T0252A – Skin on right labium  T0252B – Skin on left labium  T80000 - Vulva, labia, clitoris, and Bartholin gland  T80100 - Vulva  T80110 – Vulva, right side  T80120 – Vulva – left side  T80130 – Vulvar mucosa  T8013A – Vulvar mucosa, right  T8013B – Vulvar mucosa, left  T80200 – Mons pubis  T80210 – Labium  T8021A – Labium right  T8021B – Labium left  T80220 – Labium majus  T8022A – Labium majus, right side  T8022B – Labium majus, left side  T80230 – Commissura labiorum anterior  T80240 – Commissura labiorum posterior  T80300 – Labium minus  T8030A – Labium minus, right side  T8030B – Labium minus, left side  T80230 – Commisura labium anterior  T80240 – Commisura labium posterior  T80400 – Clitoris  T80500 – Glandula vestibularis major  T8050A – Glandula vestibularis major, right  T8050B – Glandula vestibularis major, left  T80510 – Glandula vestibularis minor  T8X110 – Cytology, vulva  T81400 – Hymen  TY4241 – Pubic region |
| Vagina | DC529 – Vaginal cancer | T81000 – Vagina  T81010 – Vaginal mucosa  T81110 – Fornix vaginae  T81115 – Top of the vagina  T8111A Fornix vaginae, right  T8111B Fornix vaginae, left  T81400 – Hymen  T81900 – Vagina and uterus  T8X210 – Cytology, vagina  T8X212 – Cytology, vaginal wall |
| Perineum and anus | DC211 – Cancer of the anal canal  DC218 – Cancer of the rectum, rectal opening, or anal canal, affecting multiple locations  DC445 – Other skin cancer of the body (anus (A), breast (B) or perineum (C)) | T00102 – Resection margin, anal  T0Y500 – Cytology, skin in perineum  T0Y507 – Cytology, skin in the anal region  T69000 – Anus  T6X920 – Cytology, anal canal  T02500 – Skin on perineum  T02507 – Skin in the anal region  T68910 – Rectum and anal canal  T69010 – Anal canal  T69015 – Anal wall  T69110 – Anal mucosa  T69120 – Anal mucosa, transitional zone  TN6890 – Neorectum and anal canal  TY1700 - Perineum  TY1701 – Anal region  TYY701 – Cytology, anal region |
| Oropharynx | DC019 – Cancer of the base of the tongue  DC024 – Cancer of tonsilla lingualis  DC051 – Cancer of the soft palate  DC052 – Cancer of the uvula  DC068 – Cancer of the oral cavity affecting multiple locations  DC069 – Cancer of the oral cavity without further specification  DC091 – Cancer of the palatoglossal arch (anterior (A) or posterior (B))  DC092 – Cancer of tonsilla palatinae  DC098 – Cancer of the tonsils affecting multiple locations  DC099 – Cancer of the tonsils without further specification  DC100 – Cancer of vallecula epiglottica  DC108 – Cancer of the oropharynx affecting multiple locations  DC109 – Cancer of the oropharynx without further specification  DC140 – Cancer of the pharynx without further specification | T51120 – Soft palate  T51130 Uvula  T51140 – Palate mucosa  T53020 – Base of tongue  T5302A – Base of tongue right side  T5302B – Base of tongue left side  T53030 – Palatoglossal arch  T5303A – Palatoglossal arch, right  T5303B – Palatoglossal arch, left  T60200 – Oropharynx  T60230 Vallecula epiglottica  T61000 – Tonsil and adenoid  T61100 – Tonsilla palatina T61110 Right tonsillae palatinaeT61120 Left tonsillae palatinaeT61130 Both tonsillae palatinaeT61140 Tongue tonsilT61150 Tonsillar archT61151 Right tonsillar archT61152 Left tonsillar archT61153 Anterior tonsillar archT61154 Posterior tonsillar archT6115A Anterior tonsillar arch, rightT6115B Anterior tonsillar arch, leftT6115C Posterior tonsillar arch, leftT6115D Posterior tonsillar arch, rightT61230 Peritonsillar tissueT6123A Peritonsillar tissue, rightT6123B Peritonsillar tissue, leftT61240 – Tonsillar level T6124A – Right tonsillar level  T6124B – Left tonsillar level  T61300 Pharyngeal tonsil |

^1^ According to Fritz APC et al. International classification of diseases for oncology (ICD-O). 3rd ed. Geneva: WHO; 2000.

^2^ SNOMED codes according to *Kodebog for patologisk-anatomiske undersøgelser. 3rd edn. .* National Board of Health, 1996.
